# Why Does General QoL Tend to Worsen Over Time Following Septoplasty Even in the Absence of Worsening Nasal Symptoms?

**DOI:** 10.1007/s12070-020-02208-x

**Published:** 2020-10-15

**Authors:** Elena Cantone, Maurizio Iengo

**Affiliations:** Department of Neuroscience, ENT section, “Federico II” University of Naples, Via Pansini, 5, Naples, Italy

**Keywords:** Septoplasty, Symptoms, Quality of life, Questionnaires, NOSE, SF-36

## Abstract

Septoplasty is one of the most frequent surgical procedures performed by head and neck surgeons. Despite surgical correction, some patients are not satisfied with their postoperative outcomes. Only a few papers report data on the long-term quality of life of patients after septoplasty, and none over 36 months. This follow up study on 68 surgical procedures aims to evaluate the differences between short term and long-term quality of life after septoplasty using a disease specific quality of life questionnaire, the NOSE, and a general health status questionnaire, the SF-36. We found a statistically significant decrease of the total long-term SF-36 scores, but we did not find changes of the NOSE scores compared with the short-term scores. These results indicated that patients perceived a worsening of their quality of life after more than 36 months following septoplasty, even in the absence of specific nasal symptoms. We could speculate that the preoperative general health performance was erroneously attributed to specific nasal symptoms, probably incorrectly emphasized or that patients did not correctly remember the preoperative clinical status. This research pointed out the importance of patients’ selection and of a thorough evaluation of their preoperative expectations before surgical planning.

## Introduction

Nasal obstruction is one the most common symptoms observed in head and neck clinical practice. It is directly related to the quality of life (QoL) and frequently caused by septal deviation. Septoplasty represents one of the most frequent surgical procedures performed by head and neck surgeons. However, despite surgical correction, some patients are not satisfied with their postoperative outcomes with consequent impairment of QoL and medicolegal problems [1–3].

Sometimes, patients complaining significant postoperative nasal obstruction may have a small residual septal deviation, whereas patients complaining mild symptoms may have a severe anatomical deformity [4]. These data suggest the presence of additional factors, independent of surgical outcomes, responsible for impaired QoL following septoplasty.

So far, only a few papers exist reporting data on the impact of septoplasty on long-term QoL [5].

The aim of this study was to evaluate the severity of long-term (after 36 months or more) symptoms following septoplasty and their impact on QoL compared with short-term scores.

## Material and Methods

This follow up study included primary septoplasties with turbinoplasty performed at ENT Departments of the “Federico II” University with a follow-up time of at least 36 months.

Patients gave their written informed consent to the study, which was fully approved by the Board of Medical Ethics of University of Naples “Federico II” (n.113/20). The study was carried out according to the Declaration of Helsinki.

All patients were identified in hospital records. Medical charts were searched for: date of surgery, gender, age, primary surgery or reoperation, surgical technique, history of nasal trauma or allergy, preoperative nasal endoscopy (NE) and active anterior rhinomanometry (AAR) [6].

Exclusion criteria were: age < 18 years, previous nasal surgery, trauma, chronic rhinosinusitis, allergy, diabetes, a history of radiation therapy to the head and neck, immunologic, heart, oncologic, malformative and neuropsychiatric diseases.

Follow up data were retrieved from the outpatient medical reports. We compared postoperative symptom relief at 6 months (T1) with that at 36–55 months (T2) using two different QoL questionnaires: the Nasal Obstruction Symptoms Evaluation (NOSE) questionnaire and the 36-Item Short Form Health Survey (SF-36) questionnaire.

The NOSE assessed disease specific symptoms, i.e. nasal obstruction, using a 4-points grading with higher scores meaning more severe nasal symptoms [7]. SF-36 questionnaire measured patients’ general health status in the previous month. It contains 36 questions that represent eight health concepts by grouping into subgroups—physical functioning (PF), role-physical (RP), role emotional (RE), bodily pain (BP), general health (GH), vitality (VT), social functioning (SF) and mental health (MH). One question was related to the change of the patient’s health status in the previous year.

Results of SF-36 were expressed as percentages and the higher the result, the better the patient’s general health [8].

During outpatient follow up subjects were also asked to report the onset of new pathologies or events that could have altered their QoL, in these cases they were excluded from the study.

### Statistical Analysis

The SPSS-PC, (version 16; SPSS Inc., Chicago, IL) was used for statistical analyzes. The Pearson’s correlation was used to determine the correlation between two numerical variables. The t-Student test was used to evaluate differences in the nasal obstruction metrics at T1 and T2. A *p* value of < 0.05 was considered statistically significant.

## Results

Sixty-eight patients [43 M, 25 F; age range 18–54 years, 28.9 ± 9.6 (mean ± SD)] met criteria for inclusion, mean follow up 44.05 ± 5.9 months. According to the medical records, before surgery the diagnosis of nasal obstruction had been confirmed by NE with a 4-mm 30-degree rigid endoscope (Storz, Tuttlingen, Germany) and AAR (Diagnostic cube, Rhino 31 Atmos Medizintechnik Lenzkirch, Germany) performed before and after decongestion under the same standard conditions, in accordance with the International Standardization Committee for Rhinomanometry [6].

Preoperative NE and AAR [total nose resistance (TNR) 0.49 ± 0.19 (mean ± SD)] confirmed the nasal obstruction due to septal deviation. Preoperative NOSE was 51.20 ± 14 (mean ± SD) and SF-36 41.54%. Surgical procedures were performed under general anesthesia by two experienced surgeons that decided and performed the operations.

Septoplasties were performed using the maxilla-premaxilla approach including a hemi-transfixion incision followed by an elevation of the septal mucoperichondrium in one side, combining mobilization, reshaping and/or removing the deviated part of the cartilage and/or by reconstruction of the septum support [5].

We performed submucous inferior turbinoplasty (without bony resection) and out-fracture of inferior turbinates [9].

We did not find statistically significant differences (*p* = 0.75) between T1 and T2 NOSE (Table 1).

**Table 1:** The comparison T1 versus T2 NOSE and SF-36 scores demonstrated a statistically significant decrease of the total SF-36 score alone in long term follow up (mean ± SD)

	T1	T2	*p*
NOSE	21.18 ± 20.1	22.2 ± 19.9	0.75
SF-36	82.4 ± 6.9	67.9 ± 3.5	0.0001*

* represents statistically significant

We did not find statistically significant differences in the T1 versus T2 NOSE in males (T1NOSE 21.5 ± 20.2 vs. T2NOSE 21.7 ± 17.6; *p* = 0.95) and females (T1NOSE 20.6 ± 20.4 vs. T2NOSE 23.2 ± 23.6; *p* = 0.70) or between males and females (T1 NOSE males 21.5 ± 20.2 vs. T1NOSE females 20.6 ± 20.4; *p* = 0.85—T2 NOSE males 21.7 ± 17.6 vs. T2NOSE females 23.2 ± 23.6; *p* = 0.77).

We did not find correlation between NOSE and age neither at T1 (*p* = 0.11) nor at T2 (*p* = 0.12).

T1 versus T2 SF-36 scores demonstrated a statistically significant decrease (*p* = 0.0001) of percentage in the long-term follow up (Table 1).

The SF-36 scores T1 versus T2 in males (T1 SF-36 55.4 ± 4.2 vs. T2 SF-36 68.3 ± 3.8; *p* = 0.0001) and females (T1 SF-36 55 ± 5.2 vs. T2SF-36 67 ± 2.8; *p* = 0.0001) showed statistically significant differences, whereas SF-36 scores between males and females at T1 (T1 SF-36 males 55.4 ± 4.2 vs. T1SF-36 females 55 ± 5.2; *p* = 0.73) and at T2 (T2 SF-36 males 68 ± 3.8 vs. T2 SF-36 females 67 ± 2.8; *p* = 0.5) did not show statistically significant gender differences.

**Fig. 1:**
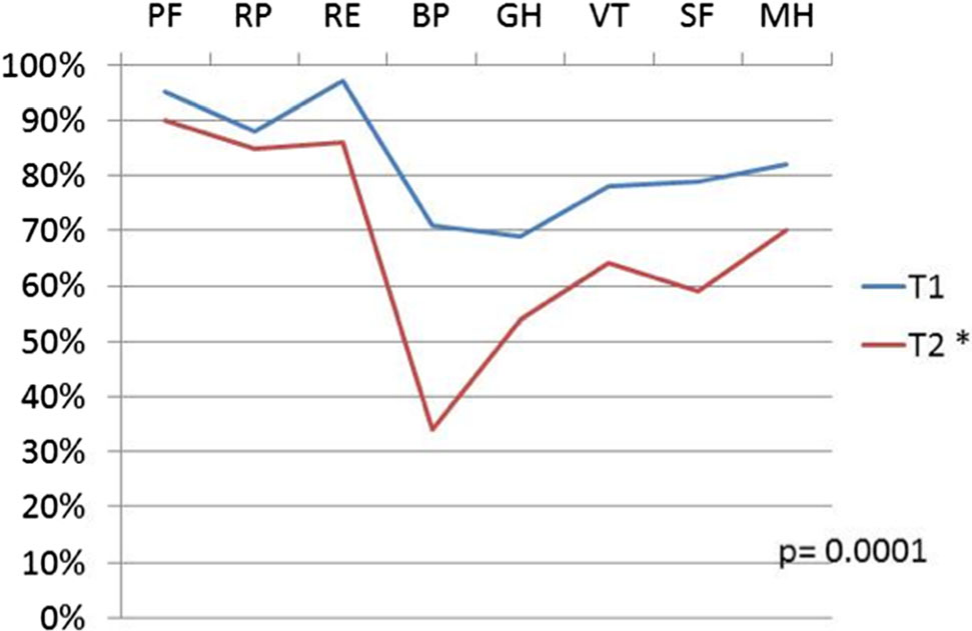
Short Form-36 (SF-36) subscores test results (%). SF-36 subscales scores at T1 were higher than at T2; *0.0001. Physical functioning (PF), role-physical (RP), role-emotional (RE), bodily pain (BP), general health (GH), vitality (VT), social functioning (SF) and mental health (MH)

There was no correlation between SF-36 and age [T1 (*p* = 0.9); T2 (*p* = 0.4)] and no correlation between NOSE and SF-36 [T1 (*p* = 0.2) and T2 (*p* = 0.06)]. SF-36 subscales scores at T1 were higher than at T2 (*p* = 0.0001), with most compromised subscales represented by BP (*p* < 0.05), GH (*p* < 0.05), VT (*p* < 0.05), and SF (*p* < 0.05) (Fig. 1).

## Discussion

Sometimes after a septoplasty the deviation can persist. Surprisingly, some persistent septal deviations are associated with patient’s satisfaction and good QoL, whereas completely restored deviations are not [1, 5]

Although previous studies have already investigated assessment, outcomes, clinical implications and patients’ satisfaction after primary septoplasty, most publications suffered from various limitations in design. Many of them were based on different surgical techniques, used only telephone follow-up surveys, relied on physician-rated outcomes rather than patient assessments of outcome, or applied either not validated outcome measures or validated outcome measures not specifically designed for nasal obstruction. In addition, previous research was based on postoperative evaluation only in the short-term or, in any case, no longer than 36 months [1, 4, 5].

Since patients, in general, tend to rate the results of their septal surgery less positively as the postoperative period gets longer, it is mandatory to evaluate surgical outcomes in the long-term [5].

Our study aims at examining the postoperative outcomes more than 36 months after septoplasty in terms of subjective assessment with the use of two QoL questionnaires, the first specifically designed for nasal symptoms (NOSE) and the second evaluating the patients’ general health status (SF-36).

We found no correlation between age at the time of surgery as well as gender, and questionnaires’ scores. Furthermore, we did not find correlation between NOSE and SF-36 scores. We found a statistically significant worsening of the total general health SF-36 score at T2 compared with SF-36 score at T1 follow up (Table 1). We also found that results were generally better among all of the eight test subgroups at short-term T1 follow up than at long-term T2 follow up (Fig. 1).

In our sample size, while the specific nasal symptomatology assessed by NOSE did not change over time, the general health status assessed by SF-36 did. These results showed that the general QoL expressed by SF-36, was perceived as worsened over time, despite the stability of the nasal-specific symptoms, as indicated by NOSE. In particular, among SF-36 subscales the most altered ones at T2 were BP, GH, VT, and SF. On the one hand, for GH, VT, SF we could think that the passing of time and the increasing age affect these subscales, on the other, for BP an explanation is rather difficult to provide.

We might suppose that the preoperative general health performance was erroneously attributed to specific nasal symptoms that were, probably, wrongly emphasized.

It must be also considered the possibility that patients did not correctly remember the preoperative clinical status.

Despite many differences among studies, all researchers showed an unsatisfactory QoL after septoplasty [10].

For this reason, it is crucial the patients’ selection and dealing with the patient’ expectations; the surgeon should make it clear to the patient as much as possible which symptoms can resolve and which cannot. Indeed, very often, the patient’s priorities are different from the surgeon’s clinical assessment. In addition, some patients could erroneously assume that any surgery will cure even not mentioned symptoms.

However, our study, demonstrated that QoL rate after septoplasty decreased with time, even after 55 months [3, 10].

## Conclusions

Our research showed that general QoL, assessed by SF-36, worsened over time, despite NOSE indicated that the nasal-specific symptoms did not. So, it is of utmost importance the patients’ selection and especially the thorough evaluation of their preoperative expectations before surgical planning.

Likewise, it is important to keep patients in a long-time postoperative follow up.
